# Preconception care practices among primary health care nurses working in public health facilities in KwaZulu-Natal

**DOI:** 10.1080/16549716.2022.2112395

**Published:** 2022-09-26

**Authors:** Winifred Chinyere Ukoha, Ntombifikile Gloria Mtshali

**Affiliations:** School of Nursing and Public Health, Howard College, University of KwaZulu-Natal, Durban, South Africa

**Keywords:** Healthcare practice, preconception care, quantitative study, primary health care, health services

## Abstract

**Background:**

Preconception care (PCC) is necessary to identify and deal with all the risk factors before conception. Some aspects of PCC, like folic acid supplementation, would be relevant to people desiring a pregnancy. Alternatively, PCC could provide contraceptive support to those with no pregnancy intention. In South Africa, primary healthcare nurses provide a comprehensive package of essential services in public health facilities to about 90% of the population at no cost. Therefore, they are the key providers of promotive, preventive, and curative services, including PCC.

**Objective:**

This study aimed to determine the level of PCC practice among primary healthcare nurses and identify determinants of effective practice.

**Methods:**

This cross-sectional descriptive survey was conducted among 196 nurses undertaking a specialisation Primary HealthCare program in a selected higher education institution. A pretested questionnaire was used to collect data that were analysed with SPSS version 27 software.

**Results:**

The overall practice of PCC was 87.8%. Older participants were significantly less likely to exhibit good PCC practice than their younger counterparts. Female participants were also less likely to have good PCC practices than their male counterparts. Married participants were significantly more likely to practice PCC than their unmarried counterparts. Participants practicing in rural areas were also less likely to have good PCC practices than their urban counterparts.

**Conclusion:**

The PCC practice of most primary healthcare nurses in the study is relatively high. The study also identified the determinants of good PCC practice that can enhance its practice. There is a need to revisit the PCC training of healthcare workers, as most indicated the need for further training.

## Background

Preconception care (PCC), according to the World Health Organisation (WHO), is the provision of various health interventions to women and couples before conception that aims at improving health by reducing environmental and lifestyle factors that might contribute to poor pregnancy outcomes [1]. This care is necessary to ensure that all risk factors are identified and addressed before conception. Some aspects of PCC, like folic acid supplementation, would be relevant to people desiring a pregnancy. Alternatively, PCC could provide contraceptive support to those with no pregnancy intention [2]. The WHO acknowledges that PCC is also feasible in middle- and low-income countries such as Sri Lanka, Bangladesh, and the Philippines [1,3]. Despite its feasibility, many African countries, including South Africa, have not effectively integrated PCC into the existing healthcare system.

In South Africa, primary health care (PHC) is delivered mainly by nurses in public health infrastructure within 5 km or more. These comprehensive basic services, which include maternal, child, and reproductive health, screening, care, and treatment of communicable and non-communicable diseases and common ailments, are provided to more than 90% of the population at no cost [4]. The PHC nurse is vital in delivering PCC to both women and men by identifying risk factors and encouraging healthy living [5]. The PHC level can deal with general and sexual health issues while enabling men’s access to services that will be a step forward to a holistic approach to men’s health [6]. An interdisciplinary healthcare provider’s collaboration and referral within a primary care setting is the most practical approach to PCC because its provision can be shared among professionals across patient referrals [7].

The latest National Confidential Committee of Enquiry into Maternal Deaths report in South Africa revealed that non-communicable diseases such as hypertensive disorders contribute to about 17.8% of maternal deaths [8]. Similarly, according to the 2017 report in Kenya, hypertensive disorders associated with pregnancy account for 15.3% of all maternal deaths in the country [9]. This percentage would reduce drastically if PCC is implemented for women of childbearing age with these risk factors [3]. PCC can support non-communicable disease prevention both in the mother and her offspring with intergenerational benefits [2]. Although the maternal and child mortality and morbidity rate in South Africa has improved compared with other countries in the region, much effort is still required to tackle the preventable causes [10].

In a position paper, the American Academy of Family Physicians urged that all family physicians provide PCC as part of family-centered health care [11]. In primary care, the clinical content of evidence-based PCC practices was developed by the Center for Disease Control and Prevention [12]. PCC incorporation is also supported in Canada [13]. Various ways of integrating PCC as a fixed protocol during maternity care have been suggested, such as postnatal check-ups, well-baby, immunization, and contraceptive services [14]. However, to maximize the delivery and uptake of preconception interventions among adolescents, a task-shifting to community health workers while liaising with communication and information technology is required. This shift will also increase PCC services integration into other programs in low- and middle-income countries [15]. The mobile phone application is a PCC delivery strategy that has received wide attention recently due to the number of people using mobile phones worldwide. Similarly, the use of reproductive life plans (RLP) to improve health outcomes among women and men of reproductive age has been identified as a strategy for delivering PCC [16].

The practice of PCC has been established in many countries such as Ethiopia, China, South Africa, Canada, and Australia [17–21]. An Iranian survey of the midwife’ practice of PCC shows that their level of provision was intermediate, and predictors of practice are age, employment status, job responsibility, and PCC knowledge [22]. Moreover, the gender of the healthcare provider may influence their practice. An Australian/New Zealand study on PCC provision among fertility specialists revealed that females are more likely to screen for eating disorders than their male counterparts; generally, few doctors routinely screen fertility patients for eating disorders during PCC assessment [23].

South Africa’s PHC reengineering has given these providers a greater responsibility in preventive care, including PCC. However, the only aspect of PCC recommendation available in the country is in the maternity and clinical guidelines for genetic services [24,25]. Some studies have been conducted in the country on general PCC for women. One study that assessed the pre-pregnancy intervention for adolescent girls found that behavioural interventions improve preconception health [26]. Another assessed the preferences of young women aged 18 to 24 for strategies to improve their preconception health and revealed that they prefer mental health interventions and printed resources compared to other interventions [27]. The latest evaluated PCC practices and utilization by healthcare providers and women at high risk of adverse pregnancy outcomes revealed inconsistent PCC practices among this group of women [20]. Factors influencing PCC utilization were mainly at the individual and institutional levels [28]. Another South African and Zimbabwean study targeted women with a history of pre-eclampsia to determine their periconceptional nutritional status. They did not start subsequent pregnancy with adequate weight and micronutrient intake [29].

Despite these mentioned studies, no study has evaluated the determinants of effective PCC practice among primary healthcare workers. PHC nurses are key in the provision of these services. However, because PHC postgraduate students are already practicing nurses in PHC facilities with exposure to PCC, and it is part of the comprehensive package of essential services, there is an imperative to establish their level of practice. Therefore, this study aimed to determine the level of PCC practice among these nurses and identify effective practice determinants. The research question asks what the current PCC practices of PHC nurses are and what predicts effective practice.

## Methods

### Operational definition

#### Preconception care

PCC implies all interventions before conception, either as advice, risk assessment, treatment, or referral. Included as PCC practices are counselling about lifestyle modification, fertility planning, screening and assessment, and interventions or referrals for management of communicable and non-communicable diseases.

### Practice

The PCC practice level of PHC nurses was measured based on how often they provide different PCC components. There are 36 items in five graded Likert-like scales (ranging from never to always), as seen in Table 2. The practice questions have a maximum score of 180. With 50% as a cutoff point, the practice level was divided into good and poor practice [17,30]. Good practice refers to those who scored 124–180 in the practice of PCC components, and poor practice refers to those who scored 36–123 in the practice of PCC components.

**Table 1.** The socio-demographic characteristics of study respondents.

Demographic data	Frequency (n)	Percentage (%)
**Age**		
24–30	73	37.2
31–40	67	34.2
>41 years	56	28.6
**Years of experience**		
1–5	85	43.4
6–10	77	39.3
>11 years	34	17.3
**Gender**		
Male	55	28.1
Female	141	71.9
**Level of education**		
Diploma	118	60.2
Bachelors	69	35.2
Masters	9	4.6
**Type of facility**		
CHC	46	23.5
PHC clinic	101	51.5
Level 1 or 2 hospital clinic	32	26.7
Others	17	8.7
**Location of facility**		
Urban	60	30.6
Semi-urban	39	19.9
Rural	97	49.5

**Table 2.** The level of preconception care practice.

PCC Counselling	Never	Rarely	Sometimes	Often	Always	M
Family planning methods	-	2.6	11.2	39.3	46.9	4.31
Pregnancy spacing	-	1.5	13.3	36.7	48.5	4.32
Physical exercise	2.6	4.1	20.9	33.7	38.8	4.02
Body weight	1.5	3.6	20.4	35.2	39.3	4.07
Nutrition	1.0	2.6	15.8	32.7	48.0	4.24
Alcohol, tobacco, and psychoactive substance use	1.5	4.1	11.7	34.2	48.5	4.24
Multivitamin containing folic acid	1.0	1.5	12.8	34.2	50.5	4.32
Importance of maintaining good control of any pre-existing medical conditions before conception	0.5	1.5	9.2	41.3	47.4	4.34
Importance of screening for STIs/HIV	0.5	1.5	8.7	30.6	58.7	4.45
Dangers of prescribed and non-prescribed medication use	-	3.6	12.8	32.1	51.5	4.32
Environmental hazards and toxins	2.0	8.7	12.8	37.2	39.3	4.03
Preventive vaccines	2.6	2.0	15.3	36.7	43.4	4.16
The importance of inviting partners for preconception counselling, risk screening, and management	2.6	7.7	11.2	36.2	42.3	4.08
**PCC Risk assessment**						
Demographic information	4.6	10.7	13.3	18.9	52.6	4.04
Past obstetric and gynecologic history	3.6	2.0	5.6	18.4	70.4	4.50
Past medical and surgical history	3.6	1.5	5.6	13.8	75.5	4.56
Genetic history or family pedigree	4.1	3.6	9.7	20.4	62.2	4.33
History of dental care/checkup	13.8	15.3	16.8	17.3	36.7	3.48
Social history, particularly lifestyle behaviours	5.1	6.6	13.8	25.5	49.0	4.07
Exposure to environmental toxins and contaminants	9.7	9.7	16.8	28.6	35.2	3.70
Pharmacologic history	5.6	7.1	7.7	35.2	44.4	4.06
Nutritional assessment, particularly BMI	3.1	3.6	10.2	29.6	53.6	4.27
Psycho-social assessment	3.6	3.1	14.3	23.5	55.6	4.24
Physical examination	2.6	2.6	6.1	25.5	63.3	4.44
Employment history	4.6	6.1	12.2	30.1	46.9	4.09
Vaccination status	3.6	3.1	11.2	33.2	96.0	4.21
**PCC Interventions and referrals**						
Folic acid supplementation/prescription	3.1	9.2	2.0	18.4	67.3	4.38
Substance use cessation: alcohol, cigarette, or other drugs	5.1	6.1	5.6	21.9	61.2	4.28
Select safe medication or substitute the existing one with a safe one	3.6	8.7	3.1	23.0	61.7	4.31
Ordering/checking routine preconception lab investigations	7.7	10.7	5.6	29.6	46.4	3.96
Diagnosing and managing acute or chronic preconception risk conditions	5.6	7.7	7.7	27.0	52.0	4.12
Controlling existing pre-gestational chronic diseases	3.1	8.2	2.6	29.1	57.1	4.29
Vaccination of client as per the national protocol	2.6	10.2	3.6	28.1	55.6	4.24
Pregnancy confirmation	1.0	7.7	2.0	21.9	67.3	4.47
Linking clients to other relevant departments or organizations	1.5	9.7	3.6	31.1	54.1	4.27
Provide nurse-initiated HIV testing and counselling	4.6	5.6	3.1	23.0	63.8	4.36

### Design and setting

This cross-sectional descriptive survey was conducted among nursing students undertaking a specialisation PHC program in a selected higher education institution.

The selected higher education institution is situated in Durban, with five campuses scattered within the province of KwaZulu-Natal [31]. The selected PHC program is a 1-year specialisation program for nurses already working in the PHC facilities. The program is decentralized into six sites for easy access to nurses.

The researchers purposively selected four study sites based on their location (urban, semi-urban, and rural). Two hundred and forty-eight PHC nurses were included in the study. Due to the manageable population, there was no need for sampling, and the entire group was invited to participate in the survey. A hundred and ninety-six respondents completed and returned their questionnaire. This number is adequate to answer the study research question according to the sample size calculation by Israel [32]. The inclusion criteria were willingness to participate in the study and working in a PHC facility for 1 year or more.

### Data collection instrument

A previously tested and validated data collection instrument, ‘*Andarg-Ethio PCC-KAP-Questionnaire for HCP’* with an acceptable Cronbach’s alpha of 0.95, was used to collect data for this study [33]. The questionnaire was initially developed in English and translated to isiZulu. The participants were allowed to choose any version they were comfortable using. The data collection instrument captured the socio-demographic factors, different aspects of PCC practices that included a 9-question general PCC practice, six items assessing what is applicable in their facilities, and the number of PCC components provided in the past month. The last aspect of practices comprised 36 items measuring PCC counselling, risk assessment, intervention, and referrals, as seen in Supplementary file 1.

Most questions in the questionnaire address actual practice, whereas some address respondents’ opinions on practice and were adapted from [34,35]. The items were on a Likert or Likert-type scale, ranging from (1) strongly disagree or never to (5) strongly agree or always. After that, the scores were added to create an overall score. There was no need for a reverse scoring as there was no negative statement. The 36-item practice was used to calculate the single practice factor categorised into two ordinal categories. Good practice refers to those who scored above 50% of the PCC practice components (124–180), and poor practice refers to those who scored 36–123 of the PCC practice components. The minimum possible score was 36, and the maximum possible score was 180. Three areas of PCC practices were assessed: PCC counselling, risk assessment, interventions, and referrals. The data collection instrument was electronically disseminated from August 2020 to March 2021 using Google Forms and a hard copy. The Cronbach’s alpha of the practice section of the current study was 0.96. The questionnaire took approximately 12 minutes to complete, and several reminders were sent to respondents to reach a good representation of the population.

### Data analysis

The collected data were entered and analysed using SPSS version 27 software. Descriptive statistics such as frequency, mean, and standard deviations were used to describe the variables. The researchers implemented inferential statistics such as binary and multiple logistic regression analytical models to determine the crude and adjusted odds ratios (COR and AOR) of PHC nurses’ PCC practice determinants. The significant level was set at P-value < 0.05.

## Results

### Socio-demographic characteristics of the study respondents

The total number of participants was 196, with a participation rate of 79%. As seen in Table 1, the PHC nurses were mainly females, 141 (79.1%); had a diploma as the highest educational qualification, 118 (60.2%); and employed in the PHC clinics’ 101 (51.5%). The largest category was in the age group of 24 to 30 years, 73 (37.2%), and the largest group had worked as PHC nurses for 1 to 5 years, 85 (43.4%).

The respondent’s level of PCC practice at their institutions, their views, training, and involvement was first assessed before their actual level of practice. The majority of the respondents, 152 (77.5), indicated that they had provided PCC services before. It is noteworthy that equal numbers of respondents agreed and disagreed on having a written PCC protocol, having seen or used a PCC protocol, and having seen national PCC guidelines. Most respondents, 175 (89.3%), agreed that they are willing to incorporate PCC into their daily practice. Almost an equal number of respondents agreed and disagreed that they had received PCC training before. The majority of the respondents, 174 (88.8%), believe they will require further PCC training. Fifty-nine percent indicated more than one professional category to be responsible for PCC in their facility, 40.3% chose only one professional out of five alternatives, but most chose PHC nurses as more suitable for PCC provision. Seventy-one percent recommend more than one category of health professionals to be responsible for providing PCC. However, the majority chose PHC nurses and doctors. They also recommended more than one setting where PCC can be provided, although most chose family planning clinics. They also suggest that PCC be provided in the clinics; they indicated HIV testing or management and chronic condition control as the primary type of PCC offered in their facilities. Twenty-four percent of the respondents stated that they did not provide any form of PCC in the past month.

### Level of preconception care practice

The respondents’ level of PCC practice was assessed using the 36-item question, which comprised their level of PCC counselling, risk assessment, intervention, and referrals. This was calculated among the 196 study participants. The respondents score ranges from 65 to 180 (M = 151.556, SD = 25.42). Fourteen respondents got the maximum score of 180, while 12.2% scored below the 50% cut-off point. Participants scored higher in PCC counselling than other practice groups, with all mean scores above 4.00. PCC counselling items that scored higher than others were counselling about the importance of screening for STIs/HIV (M = 4.45, SD = .760), importance of maintaining good control of any pre-existing medical conditions before conception (M = 4.34, SD = .750), and pregnancy spacing (M = 4.32, SD = .760). Those items that respondents scored low in were counselling about physical exercise (M = 4.02, SD = .997), environmental hazard and toxins (M = 4.03, SD = 1.027), body weight (M = 4.07, SD = .936), and the importance of inviting a partner for preconception risk screening and management (M = 4.08 SD = 1.035).

PCC risk assessment items that scored higher than others were assessment of past medical and surgical history (M = 4.56, SD = .940), past obstetrics and gynecologic history (M = 4.50, SD = .958), and physical examination (M = 4.44, SD = .913). Assessment of the history of dental care or checkup (M = 3.48, SD = 1.459) and exposure to environmental toxins and contaminants (M = 3.70, SD = 1.303) scored low.

The PCC interventions and referral items that got high scores were pregnancy confirmation (M = 4.47, SD = .936), provision of nurse-initiated HIV testing and counselling (M = 4.36, SD = 1.088), and folic acid supplementation/prescription (M = 4.38, SD = 1.096). Ordering or checking routine preconception lab investigations was the only item that scored low among this group (M = 3.96, SD = 1.283).

### Determinant of good preconception care practice

A multivariate logistic regression analysis was used to assess the determinants of good PCC practices among PHC nurses in KwaZulu-Natal. The PCC practices’ determinants were age, experience, gender, ethnicity, education level, marital status, and facility location. Table 3 summarises the unadjusted and adjusted odds ratio for PCC determinants. The odds ratio is presented at a 95% confidence interval and the corresponding significance value (p-value < 0.05).

**Table 3.** Logistic regression analysis showing determinants of good preconception practice among primary healthcare nursing students.

Factor	COR (95% CI)	P-value	AOR	P-value
**Age**				
24–30 *(Ref)*	1		1	
31–40	0.89 (0.46–1.72)	0.719	0.89 (0.35–2.25)	0.805
>41 years	0.53 (0.26–1.08)	0.081	0.23 (0.07–0.75)	0.016
**Gender**				
Male *(Ref)*	1		1	
Female	0.67 (0.36–1.26)	0.217	0.46 (0.22–0.97)	0.041
**Level of education**				
Diploma (Ref)	1		1	
Bachelors	0.83 (0.46–1.51)	0.544	0.59 (0.28–1.23)	0.158
Masters	0.33 (0.61–1.25)	0.175	0.17 (0.03–1.08)	0.060
**Marital status**				
Single (*Ref)*	1		1	
Married	1.20 (0.66–2.18)	0.560	2.62 (1.14–6.03)	0.023
Divorced	1.82 (0.46–7.15)	0.390	4.85 (0.93–25.36)	0.061
**Location**				
Urban (*Ref)*	1		1	
Semi-urban	2.44 (1.06–5.65)	0.037	1.57 (0.57–4.34)	0.387
Rural	0.63 (0.33–1.22)	0.170	0.32 (0.13–0.78)	0.013
**Ethnicity**				
Other *(Ref)*	1		1	
Black	0.60 (0.22–1.58)	0.299	0.86 (0.25–2.88)	0.801
**Years of experience**				
1–5 *(Ref)*	1		1	
6–10	0.61 (0.33–1.13)	0.118	0.60 (0.24–1.42)	0.242
>11 years	0.63 (0.24–1.50)	0.301	0.54 (0.14–2.06)	0.364

P Value < 0.05; CI, confidence interval; COR, Crude Odd Ratio; AOR, Adjusted Odd Ratio

Compared to their counterparts between 24 and 30 years, the odds ratio for nurses aged 40 and above was significantly reduced. This might suggest that older women are less likely to have good PCC practices. Though not significant, the odds ratio for the nurses with six or more years of experience reduced compared to their less experienced counterparts. Surprisingly, this study found that female nurses are significantly less likely to have good PCC practices when compared to their male counterparts. Though not significant, black nurses are less likely to have good PCC practices than other ethnic groups.

Nurses with master’s degrees are less likely to have good PCC when compared to their counterparts with diplomas. Marital status is associated with PCC, with married nurses significantly more likely to have good PCC when compared to single/unmarried nurses. Divorced nurses are also more likely to have good PCC when compared to their single counterparts. The study also found that nurses from rural locations/facilities are significantly less likely to have good PCC practices than their urban counterparts.

## Discussion

The PCC practice level was poor among 12.2% of the respondents and good among 87.8%. Therefore, in the current study, the PCC practice level among most PHC nurses is good. This is much higher than that of an Ethiopian survey where only 15% provided PCC (Kassa et al., 2019) and an Iranian study where healthcare practitioners’ PCC practice was reported to range from 25 to 65% [36]. The finding is also inconsistent with a Netherlands study where PHC providers were reported to be underserving women regarding PCC provision [35]. Associations were found between the participant’s demographic variables such as age, gender, marital status, and location of practice with their PCC practice. The results suggest that good PCC practices are reduced with age, perhaps due to their decreased ability to give birth or the type of professional education they received. The reason for females being less likely to provide PCC is not known because it is generally expected to be the opposite. The influence of the practice location on their practice level might be attributed to the nature of their duties at the facility and the type of patients seen in these settings. This has an implication for practice and suggests areas where effort is needed. A recent scoping review of the articles on PCC in Sub-Saharan Africa also revealed that age and location of practice were associated with PCC knowledge but not provision among healthcare workers [37].

Although most respondents (77.5%) reported providing PCC services to women, most had not offered any form of PCC in the preceding month. Those who had recently provided PCC rendered the service less than three times in the prior month. The percentage of nurses in the current study who had never offered PCC is higher than in a Nigerian study [38]. The findings are comparable with a study in the Netherlands, where only 20% of midwives reported having rendered PCC consultation in the prior 2 months [35]. The availability of PCC resources for women and healthcare workers has been considered a prerequisite to PCC provision [39]. Having a written PCC protocol, having seen or used a national PCC guideline drew mixed responses. This is similar to a study among general practitioners in Australia and New Zealand, where nearly half of the respondents were aware of the existence of a PCC guideline [40]. They also demonstrated their willingness to incorporate PCC into their daily practices. This is comparable to a study among doctors and nurses in Nigeria, where 91.2% were willing to offer PCC [38].

Healthcare providers often report not being adequately prepared for PCC provision due to insufficient training [38,41]. In the current study, respondents reported mixed responses regarding their PCC training; nevertheless, most indicated that they would require some PCC training. This is contrary to an Australian study’s findings where only 3% of the respondents indicated a lack of appropriate knowledge and therefore expressed a desire for further PCC training [18]. Practitioners vary when recommending which healthcare provider should be responsible for PCC provision. In Australia and New Zealand, general practitioners feel that they have to provide PCC as the primary care setting renders services to about 80% of women of childbearing age [40]. The same situation prevails with midwives; although having less access to non-pregnant women of childbearing age, most are willing to provide PCC [14,22]. In this study, the PHC nurses were indicated to be responsible for PCC in the facilities of most respondents. Most respondents also recommended them as the provider of PCC.

Various settings with their strengths and weaknesses have been identified for effective integration of PCC. These include primary care, hospital-based care, dedicated clinics, and high-risk care [42]. Berglund and Lindmark [43] argue that PCC should be integrated into family planning and sexually transmitted infection services as contraceptive counselling is not solely to prevent unintended pregnancy but also to safeguard fertility until pregnancy is desired. The participants thought that family planning clinics were a good place for PCC. In Sweden, nurse midwives are responsible for about 90% of family planning provisions [44]. In both developed and developing countries, RLP has been proven to enable midwives and other healthcare workers to improve the quality of contraceptive counseling and support women’s reproductive goals [45,46]. An ideal PCC visit involves an aspect of education, risk assessment, and intervention if necessary [42,47]. Therefore, among the three areas of PCC practices assessed, respondents got higher scores in the area of PCC counselling. Obesity reduction and management is another area that requires urgent attention. Although obesity can be prevented to reduce adverse pregnancy outcomes [48], combatting this must start early in life. Obesity is linked to hypertension, contributing to 18% of maternal deaths in South Africa [8]. Nurses can have an essential role in PCC. However, they must first have a good PCC education and be motivated.

In the item of PCC risk assessment, most of them got a high score on the evaluation of past medical and surgical history, obstetric and gynaecological history, and physical examination. This corroborates the findings among general practitioners where past medical and obstetric history were among the most regularly discussed PCC components [40]. A review of preconception health interventions revealed that exposure to harmful environmental contaminants is largely neglected [49]. In the same vein, the participant nurses were lacking in the category of dental care or check-ups and exposure to environmental toxins and contaminants. The study respondents were also not enthusiastic about assessing women’s oral health. However, it has been proven that good oral health of the mother during pregnancy improves both oral health outcomes and pregnancy outcomes of mother and baby [50].

Respondents’ scores for PCC interventions and referrals were high in pregnancy confirmation, provision of nurse-initiated HIV testing and counselling, and folic acid supplementation or prescriptions. Nurse-initiated and managed antiretroviral therapy is an aspect of PCC for people living with HIV that nurses in Sub-Saharan African countries are very familiar with [51]. Provision and uptake of safer conception strategies is an extraordinarily researched area of PCC [52,53]. Nyapaver and Arbour [54] illustrated the ease with which some PCC interventions and laboratory investigations can be included in any visit with a woman of childbearing age to meet her needs. The study respondents were good at ordering or checking routine preconception laboratory investigations.

## Conclusion

Most PHC nurses have an excellent PCC practice level. However, not all PCC components are given equal attention. Most PCC areas that pertain to the South African disease profiles were well attended to, except for the aspect of obesity. The PHC nurses should be aware of the growing danger of obesity in society, especially among women of childbearing age, and act accordingly. The nurse’s age, gender, marital status, and location of practice were statistically associated with the practice of PCC. We recommend the utilisation of RLP in primary care to improve the quality of contraceptive counselling. There is also the need for educational campaigns to increase the PCC awareness and utilisation of women. The extent of PCC training among PHC nurses should be investigated and improved.

The study may be underpowered due to the small sample size; therefore, the results must be considered cautiously. Another limitation may be due to the design of this study; recall bias cannot be ruled out. Furthermore, this study was carried out on PHC nurses undergoing a postgraduate specialisation program, so there is a possibility that the study environment that they were in may have influenced their knowledge and practice. Knowledge does not necessarily translate into practice, especially among healthcare practitioners in primary care who are limited by time and resources, as seen in other studies [36]. Thus, considering what could enable PCC translation in the South African context is highly recommended.

## Paper context

The Centers for Disease Control and Prevention identified the need for studies on the factors that result in variations in the healthcare worker’s provision of PCC. PCC assists in the reduction of maternal and child mortality. The degree of attention given to different PCC clinical content is different. Several demographic factors influenced PCC provision among healthcare workers. Preconception training of healthcare workers should be carefully considered.
